# N, S-Codoped Activated Carbon Material with Ultra-High Surface Area for High-Performance Supercapacitors

**DOI:** 10.3390/polym12091982

**Published:** 2020-08-31

**Authors:** Qinghua Yuan, Zhuwen Ma, Junbiao Chen, Zhenrui Huang, Zeming Fang, Peng Zhang, Zhidan Lin, Jie Cui

**Affiliations:** 1 Guangdong Provincial Engineering & Technology Research Center for Tobacco Breeding and Comprehensive Utilization, Crops Research Institute, Guangdong Academy of Agricultural Science, Guangzhou 510640, China; qinghuay@foxmail.com (Q.Y.); conghuama@163.com (Z.M.); Jbchen2007@126.com (J.C.); 2 Institute of Advances Wear & Corrosion Resistant and Functional Materials, Jinan University, Guangzhou 510632, China; tzhangpeng@jnu.edu.cn (P.Z.); linzd@jnu.edu.cn (Z.L.); 3 Analytical and Testing Center, South China University of Technology, Guangzhou 510640, China; czcuijie@scut.edu.cn

**Keywords:** supercapacitor, carbon material, hetero atom doping, activated carbon, biochar, tobacco

## Abstract

The recycling of macromolecular biowastes has been a problem for the agriculture industry. In this study, a novel N, S-codoped activated carbon material with an ultrahigh specific area was produced for the application of a supercapacitor electrode, using tobacco stalk biowastes as the carbon source, KOH as the activating agents and thiourea as the doping agent. Tobacco stalk is mainly composed of cellulose, but also contains many small molecules and inorganic salts. KOH activation resulted in many mesopores, giving the tobacco stem-activated carbon a large specific surface area and double-layer capacitance. The specific surface area of the samples reached up to 3733 m^2^·g^−1^, while the maximum specific capacitance of the samples obtained was up to 281.3 F·g^−1^ in the 3-electrode tests (1 A·g^−1^). The doping of N and S elements raised the specific capacitance significantly, which could be increased to a value as high as 422.5 F·g^−1^ at a current density of 1 A·g^−1^ in the 3-electrode tests, but N, S-codoping also led to instability. The results of this article prove that tobacco stalks could be efficiently reused in the field of supercapacitors.

## 1. Introduction

Environmental protection is vital in modern society. Transforming wastes into resources and applying clean energy are two important ways to protect the environment. A great number of biowastes are produced in agriculture and industry, like stalks, peels and sawdust. The main components of these biowastes are cellulose, hemicellulose and pectin [1]. Biowastes are usually biomass macromolecules mainly composed of C, H, O and N so that they can be pyrolyzed into biochar by high-temperature heat treatment in an inert atmosphere. The carbon-rich residue is called biochar when other atoms are removed at high temperatures. In recent years, biowastes have been recycled to produce biochar for novel energy storage systems such as electrode materials and conductive fillers, which greatly benefit both the recycling of wastes and the promotion of renewable energy.

Among energy storage devices, supercapacitors are particularly useful due to their higher power density and longer cycle life [2]. Supercapacitors can be divided into electric double layer capacitors (EDLCs), pseudocapacitors and hybrid capacitors, according to the energy storage mechanism. Although pseudocapacitors, which mainly store energy by fast redox reaction, are receiving increasing attention for their higher energy density, their poor cyclic stability limits their practical application, and therefore the most used supercapacitors are still EDLCs. Since EDLCs store energy mainly via the electric double layers between electrodes and electrolyte, activated carbon materials with a high specific area have become the most commonly used electrode materials [3]. Different kinds of biomass wastes have been made into activated carbon materials for supercapacitors, like garlic skin [4], pepper [5] and coconut shells [6].

However, pure activated carbon materials still suffer from several weaknesses. An excessive increase of the surface area causes a low volumetric energy density; chemical inertness and hydrophobicity result in a poor interaction between nanocarbons and aqueous electrolytes; and the low number of delocalized electrons from the conduction band per atom limits the theoretical capacitance of EDLCs. An effective method to solve these problems is heteroatom doping, which could simultaneously tailor the fundamental properties and improve the electrochemical properties [7].

Tobacco wastes are one of the most urgent agricultural residues to need proper disposal. Although considered harmful, the tobacco industry is still the primary source of revenue in many regions. For example, the China Statistical Yearbook-2019 shows that in 2018 China produced 2.24 × 10^6^ tons of tobacco leaves [8]. Most tobacco biowastes are directly burned or buried onsite when tobacco leaves are harvested, which may lead to potential contamination and pollution because of the toxin substances inside the tobacco biowastes.

Some new disposals for tobacco wastes have recently been developed, such as composting them [9], cocombustion with other fuels [10,11] and transforming them into activated carbon for contaminant adsorption [12,13,14]. However, studies that use tobacco biowastes for energy storage are still rarely seen.

Kleszyk et al. [15] found that the high potassium content of the tobacco stem caused self-activation at high temperatures, leading to a highly microporous carbon with a narrow pore size distribution, which greatly enhanced the electrochemical capacitance. Zhong et al. [16] prepared hierarchical porous carbon material for a lithium-sulfur battery via facile pyrolysis of the tobacco stem. N, O, S tridoping happened in the heat treatment because of the biomass content inside the tobacco stem, which was very efficient in entrapping polysulfides via the synergistic effect of the structural confinement and chemical barrier in a Li-S battery. Zhao et al. [17] prepared a novel tobacco rods-derived carbon (TC by hydrothermal carbonization and KOH activation strategy for supercapacitors). The electrochemical properties of the samples derived from different varieties of tobacco leaves were compared, and the optimized specific capacitance of the samples reached 286.6 F·g^−1^ at a current density of 0.5 A·g^−1^.

Still, such studies remain narrow in focus, dealing only with tobacco leaves and stems, and there are relatively few studies on tobacco stalks in the area of activated carbon material. As the amount of straw waste produced in the tobacco industry is a lot larger than that of tobacco stems, it is necessary to study the disposal of tobacco stalks.

This study aims to explore the feasibility of using tobacco stalks as a high-performance electrode material for supercapacitors. Using tobacco stalks as the carbon source, a novel N, S-codoped activated carbon material with an ultra-high specific area was prepared by KOH activation and thiourea doping. The effects of KOH activation and N, S-codoping on the carbon materials’ structure and performance were investigated. The results show that, compared with other activated carbon materials, the products obtained in this study possess a higher specific area when applying the same chemical activating agents and show an excellent electrochemical capacity. This study confirms that the tobacco stalk is a potential candidate in the production of high-performance activated carbon materials, and that the research value of tobacco stalks is as high as that of other tobacco wastes.

## 2. Materials and Methods

### 2.1. Materials

Tobacco stems of the 8I-26 were obtained from the tobacco farms in Nanxiong City (Guangdong, China). Other reagents that were used mainly included KOH (Chemical Reagent Factory, Guangzhou, China), Thiourea (Damao Chemical Reagent Factory, Tianjin, China) and Hydrochloric acid (Damao Chemical Reagent Factory, Tianjin, China).

### 2.2. Preparation of Activated Tobacco Carbon (ATC)

The tobacco stalks were washed with clean water and then air-dried. The roots, leaves, stems and epidermal layer were removed before the stalks were crushed into 50–200 mesh powder. The powder was further dried in an oven at 120 °C for 24 h. The stalk powder was then placed in a tubular furnace and heat-treated at 550 °C for 2 h at a heating rate of 5 °C min^−1^ under a nitrogen atmosphere (the flow rate was 3 mL/min) and naturally cooled in the furnace. The residual carbon precursors were mixed with KOH at a mass ratio of 1:1, 2, 3, 4 by grinding with the assistance of a small amount of water. The obtained slurry was dried under vacuum at 80 °C for 12 h to obtain the C/KOH mixture.

The mixture was put in a nickel boat and had another heat treatment at 850 °C for 2 h at a heating rate of 5 °C min^−1^ under a nitrogen atmosphere (the flow rate was 3 mL·min^−1^). The resultant products were washed thoroughly with 1 M HCl solution to remove the excessive KOH and other byproducts like inorganic salts, followed by filtration. The filtered materials were diluted by deionized water and dried in the vacuum drying oven at 120 °C for 12 h. The obtained samples were noted as ATC-X, where X means the mass ratio of the carbon precursor and KOH. For comparison, the samples were also prepared using the tobacco leaves and stems, following the same steps (mass ratio of KOH and precursor = 3:1).

### 2.3. Synthesis of N, S-Codoped Activated Carbon (NS-ATC)

Thiourea, the nitrogen and sulfur source, was resolved in deionized water and then mixed with ATC-3 at a mass ratio of 1:2, 3, 4, after which it was thoroughly stirred for 2 h. The dispersion was dried at 80 °C to obtain the thiourea/ATC-3 mixture, which was then heat-treated at 800 °C at a heating rate of 5 °C min^−1^ under a nitrogen atmosphere (the flow rate was 3 mL·min^−1^) and held for 1 h. The obtained products were sufficiently washed with deionized water and dried at 80 °C for 24 h. The obtained samples were noted as NSATC-Y, where Y means the mass ratio of ATC-3 and thiourea.

### 2.4. Characterization

The morphology and microstructures of the samples were observed by field-emission scanning electron microscopy (FESEM, Zeiss ULTRATM 55, Oberkochen, Germany) and field-emission transmission electron microscopy (FETEM, JEOL JEM-2100F, Tokyo, Japan). The FESEM was equipped with an energy dispersive spectrometer (EDS, BRUKER XFlash 6I30, Billerica, MA, USA), which was used to analyze the element distribution.

A thermogravimetric analysis (TGA, Mettler Toledo TGA/DSC 3+, Zurich, Switzerland) was done to analyze the decomposition process of each component. Furthermore, the pore size distribution and apparent surface area of the samples were obtained by N2 adsorption/desorption isotherms using a BeiShiDe 3H-2000PS2 (Beijing, China) surface area porosity analyzer. X-ray diffraction (XRD) patterns of the carbon samples were obtained on a Rigaku Ultima IV instrument (Tokyo, Japan) at 40 kV/20 mA using a Cu target. An x-ray photoelectron spectroscopy (XPS, Escalab250Xi, Thermo-VG Scientific, Waltham, MA, USA) technique was conducted using an Al Kα radiation (1486.6 eV), and a Raman spectroscopy (HORIBA LabRAM HR Evolution, Minami-ku Kyoto, Japan) analysis was performed to further confirm the possible phases and compositions of the samples. To investigate the inorganic elements of the tobacco stalk, tobacco stalk powder was burned to ashes under air atmosphere at 1000 °C in a tubular furnace before EDS tests.

The electrochemical properties of the samples were characterized via galvanostatic charge/discharge (GCD), cyclic voltammetry (CV) and electrochemical impedance spectroscopy (EIS) using a CHI 760E workstation (CH Instruments, Austin, TX, USA).

The tests were mainly performed in a three-electrode cell in a 6 M KOH solution at room temperature, with a Pt sheet of 10*10 mm as a counter electrode and a saturated calomel electrode as a reference electrode (SCE). To prepare the working electrodes, the carbon samples were ground into powders and mixed with a polytetrafluoroethylene (PTFE) binder and acetylene black in a weight ratio of 80:10:10, after which the mixture was pressed onto a nickel foam. The two-electrode test was conducted in a solid-state capacitor. The working electrode was prepared in the same way as described before, and a polyvinyl alcohol (PVA)/KOH gel electrolyte was used. PVA (2.5 g) was dissolved in 20 mL of DI water at 90 °C under vigorous stirring. After the PVA aqueous solution cooled down, KOH solution (10 mL with 3 g of KOH) was added dropwise with continuous stirring.

For the three-electrode test, the gravimetric specific capacitance of the samples can be obtained according to Equation (1):

(1) $$C_{s}=\frac{I\Delta t_{1}}{m_{1}\Delta V}$$

where *C_s_* (F·g^−1^) is the gravimetric specific capacitance of the working electrode, *I* (A) is the current, Δ*t*_1_ (s) is the discharging time, *m*_1_ (g) is the mass of active material on the working electrode and Δ*V* (V) is the range of the potential.

For the symmetrical supercapacitor, the specific capacitance (*C_cell_*, F·g^−1^), energy density (*E*, Wh·kg^−1^) and power density (*P*, W·kg^−1^) are calculated by the following equations:

(2) $$C_{\mathrm{cell}}=\frac{I\Delta t}{m_{2}\Delta V}$$

(3) $$E=\frac{C_{\mathrm{cell}}{\Delta V}^{2}}{2\times 3.6}$$

(4) $$P=\frac{3600E}{\Delta t}$$

where *C_cell_* (F·g^−1^), *m*_2_ (g), *I* (A), Δ*t* (s) and Δ*V* (V) correspond to the capacitance of the cell, total mass of the active material on the two electrodes, current, discharging time and potential range, respectively.

## 3. Results and Discussion

### 3.1. Morphology and Structure of ATC-X Materials

First, the thermogravimetric analysis of the tobacco stalk was conducted (Figure S1). The thermal decomposition of the sample can be divided into three stages. The first stage involves water evaporation below 200 °C, and the second is a sharp decline between 220–370 °C, attributed to the devolatilization of organic tissue. The third is the slow decline of the weight after 400 °C, which can be regarded as the carbonization process [18]. Because the weight of the sample did not decrease markedly over 500 °C, the preparation temperature of the precursors was set at 550 °C. Chemical activation is often used to produce porous carbon material. The pore structure and specific areas of materials are influenced by temperatures and the mass of activating agents. A relatively high temperature is required to develop a highly porous structure, so the activation temperature was set to be 850 °C [19]. The influence of the mass ratio of KOH and precursor was investigated in relation to the morphology, structure and electrochemical performances.

**Figure 1:**
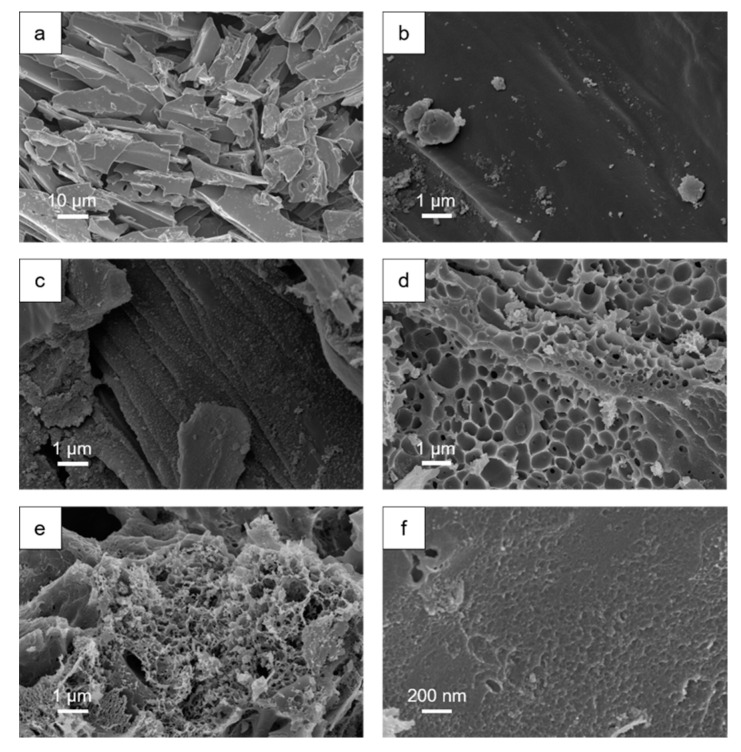
(**a**) SEM image of TC precursor; (**b**) SEM image of ATC-1 sample; (**c**) SEM image of ATC-2 sample; (**d**) SEM image of ATC-3 sample; (**e**) SEM image of ATC-4 sample; (**f**) high-magnification SEM image of ATC-3.

Figure 1 shows the scanning electron microscopy (SEM) images of the samples. The nonactivated carbon precursor TC-550 (Figure 1a) presented a flaky, smooth surface with a length and width of several micrometers to several tens of micrometers, as well as a thickness of about 1~2 μm, which maintained the original shape of the crushed stalk powder on the whole. The ATC-1 (Figure 1b) and ATC-2 (Figure 1c) samples largely retained the lamellar structure of the precursor, but it could be observed that the surface became rough, with many microscopic pores. On the other hand, the ATC-3 sample showed many large pits on the sheet surface (Figure 1d). In addition to the large micron-sized pits, there were also a large number of microscopic pores, like for ATC-1 and ATC-2 (Figure 1f). When the KOH:TC ratio rose to 4:1, the structural integrity of the ATC-4 sample was damaged, and the original lamellar structure was lost (Figure 1e). Maintaining the original microscopic sheet structure of the sample could help avoid the agglomeration of the material in use, while the structural damage might also cause problems such as a decline of electrical conductivity and ion transportation.

These porous structures are caused by two kinds of activation effects of KOH. Between 400–600 °C, the reaction of KOH and carbon materials in the heating process can be written as follows [20]:

(5) $$6\mathrm{KOH}+2C\to {2K}_{2}{\mathrm{CO}}_{3}{+2K+3H}_{2}$$

When KOH is totally consumed, and the temperature rises to more than 700 °C, some more extra reactions happen. These reactions etch the surface of the bulk carbon material, causing a large number of mesopores.

(6) $$K_{2}{\mathrm{CO}}_{3}\;\to {\;K}_{2}{O+\mathrm{CO}}_{2}$$

(7) $${\mathrm{CO}}_{2}+C\to 2\mathrm{CO}$$

(8) $$K_{2}{\mathrm{CO}}_{3}+2C\to 2K+3\mathrm{CO}$$

(9) $${C+K}_{2}O\to 2K+\mathrm{CO}$$

Besides, since the boiling point of K is about 800 °C, when the temperature exceeds its boiling point, K vapor will drill into the layers of carbon and cause damage to the structure of the carbon material. In addition, H_2_O and CO_2_ produced in the activation process also play a role in the physical activation of carbon.

**Figure 2:**
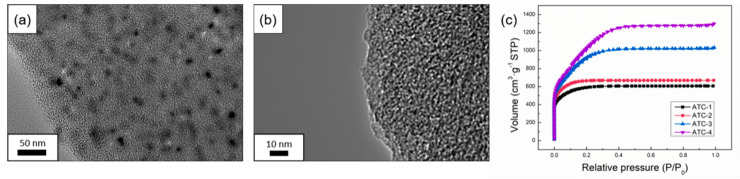
(**a**,**b**) TEM image of ATC-3; (**c**) N_2_ adsorption/desorption isotherms of ATC-1/2/3/4 at 77 K.

Since the pores are too small to observe by SEM, TEM and a N2 adsorption–desorption test were conducted to further analyze the microstructure of ATC-X samples. TEM images of the ATC-3 sample showed that the sample contained many mesopores inside (Figure 2b). It is worth noting that nanosized dots could be found in the image (Figure 2a), which are believed to be the result of the complex and diverse pyrolysis mechanism of different components of biomass material [16].

The pore structure of the samples was measured by a N_2_ isothermal adsorption/desorption test at 77 K. It can be seen from Figure 2c that the isotherms of the ATC-X samples showed typical Type-I characteristics. The adsorption curves of the four groups of ATC-X samples all showed a vertical rise in the low-pressure region (P/P0 < 0.1) and reached the saturated adsorption after a continuous rise, which means that the nitrogen gas was mainly adsorbed by micropores at a low pressure. Moreover, the desorption curves overlapped with the adsorption curves without obvious hysteresis loops, indicating that the samples had a typical porous microstructure [21], like most existing activated carbon products. At a near-saturation pressure (P/P0 = 0.99), the curve of the ATC-4 sample moved upward slightly, which was due to the coalescence of nitrogen.

The specific areas of the samples were calculated by BET method. Consistent with the result of SEM, the ATC-X samples had a high specific area, which increased with an increase of the KOH content. The specific area of the ATC samples was 1910, 2095, 3177 and 3733 m^2^·g^−1^ when the mass ratio of KOH on precursor was 1, 2, 3 and 4, respectively. In summary, the samples obtained in this paper have an ultra-high specific surface area.In general, activated carbon materials converted from tobacco waste tend to have a higher specific surface area [17,22], and the same applies for tobacco stalk. This may be attributed to the high content of metal elements in the bio tissue, which have an auxiliary effect in the activation [23]. To prove this hypothesis, an EDS test was performed to investigate the content of elements of tobacco stalk ash and TC-550 precursors (Table 1). After the tobacco stalk was burnt out, the ash that remained contained 25.17% of potassium, and the potassium content of TC-550 precursor was also up to 2.19%. These naturally embedded elements could be considered as activation agents with a molecular dispersion in the carbon precursor, leading to a self-activation effect, which has been reported in the case of tobacco stems [15]. Compared with other porous carbon materials obtained by similar methods and reported in the literature, the ATC-X samples prepared in our study boast markedly larger specific areas, and the comparison was listed in Section 3.2 together with the results of electrochemical characterizations (Table 2).

**Figure 3:**
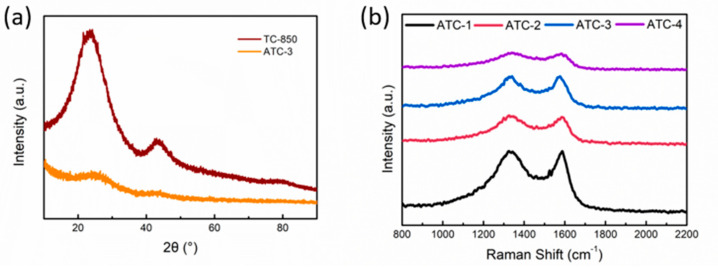
(**a**) XRD spectra of TC-850 and ATC-3; (**b**) Raman spectra of ATC-1/2/3/4.

In the XRD spectra of TC-850 (Figure 3a), there are two broad peaks at about 26° and 44°, like for most biomass carbon material, indicating a typical amorphous carbon structure with a small amount of graphitization. The intensity decreases drastically after KOH activation, suggesting the destruction of the carbon material’s structure. The structural characteristics of the ATC samples were further studied by Raman spectroscopy (Figure 3b). The D peak (~1350 cm-1) indicates a disordered structure of carbon, which is caused by defects and impurities, while the G peak (~1580 cm-1) is the characteristic peak of a layered graphite structure [24]. Therefore, the intensity ratio of these two peaks, I_D_/I_G_, reflects the degree of graphitization of the sample to some extent. It can be seen that with the increase of the amount of KOH, the width of the D peak of the sample increases, and the I_D_/I_G_ value also increases. However, with the increase of the amount of KOH, the peak intensity of the Raman spectrum of the sample decreases greatly, which indicates that the structural integrity of the sample is weakened and is easier to destroy by laser.

### 3.2. Electrochemical Performance of ATC-X Materials

Since the ATC-X samples have ultra-high specific areas, they are expected to have good electrochemical performances. Basic electrochemical characterizations were conducted in a three-electrode system.

**Figure 4:**
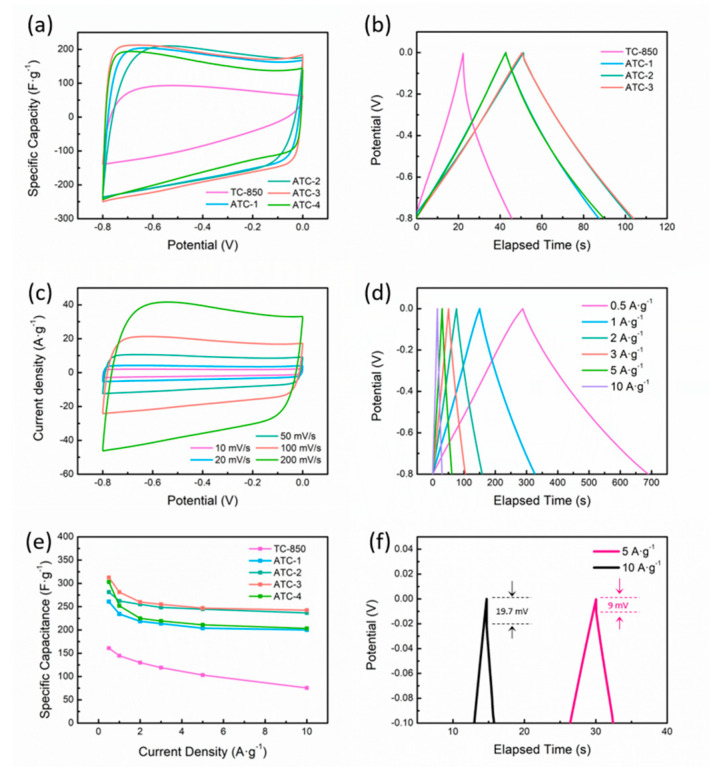
(**a**) CV curves of samples with and without activation at 50 mV·s^−1^; (**b**) GCD curves of samples with and without activation at 3 A·g^−1^; (**c**) CV curves of ATC-3; (**d**) GCD curves of ATC-3 at different current densities; (**e**) Specific capacitance of samples at growing current densities; (**f**) IR drop at high current densities.

Figure 4a shows the CV curves of the TC-850 (control group) and ATC-X (experiment group). The shape of the CV curves of TC-850 is more similar to a triangle than to a rectangle, indicating the ion-sieve effect of samples caused by the lack of mesopores, while the quasi-rectangular CV curves of the ATC-X samples clearly prove the EDLC behavior of the porous carbon materials. The CV curves gradually turn into spindle shapes but still stay centrosymmetric at a higher sweeping rate. The improved shape and higher area of the CV curves prove that the KOH activation could greatly enhance the electrochemical performance of the tobacco-based biochar. More details on the CV test can be seen in Figure S2.

However, the effect of the KOH content on the electrochemical performance still needs to be investigated. Specific capacitances at different current densities were calculated using Equation (1). It can be seen that the GCD curves of the samples are all similarly linear (Figure 4b), showing a typical EDLC behavior. The specific capacitances of the control group TC-850 (Figure S3) were calculated to be 160.9/144.7/130.01/119.1/103.1/75.6 F·g^−1^ at 0.5/1/2/3/5/10 A·g^−1^.

In addition, at lower current densities, the GCD curves show a slight deviation from linearity at a lower negative potential (Figure 4d), indicating that the hydrogen evolution reaction (HER) has taken place [25]. At higher current densities, an IR drop caused by internal resistance can be noticed (Figure 4f). To avoid the influence of the IR drop and side reaction, only the middle part of the discharge curves (−0.2 V–−0.6 V) is taken into account for calculation, and the correlation formula is formula 2–6. The attenuation of the specific capacitance of each sample with the increase of the current density before and after activation is summarized as shown in Figure 4e.

The electrochemical performance of tobacco stem-activated carbon increases at first and then decreases with the increase of the amount of KOH, and the ATC-3 samples show the highest specific capacitance in the test range. The specific capacitance of the ATC-3 sample is calculated to be 312.5, 281.3, 260.0, 255.0, 246.9 and 242.5 F·g^−1^ when the current density is 0.5, 1, 2, 3 and 5 A·g^−1^, respectively. Compared with the specific capacitance at 0.5 A g^−1^, the specific capacitance retention is 77.6% at 10 A·g^−1^, showing a great rate capability. The Nyquist curves of all activated sample show a similar shape (Figure S3), with a semicircle in the high-frequency region and a near vertical line in the low-frequency region, which is a typical impedance behavior of activated carbon materials.

When the ratio of the KOH is relatively low, the specific capacitance of the sample increases with the amount of KOH, mainly because increasing the amount of KOH could enlarge the specific surface area of activated carbon materials and therefore improve the electrochemical properties. However, the specific surface area of the ATC-4 sample is the largest, but the specific capacitance is lower than that of the ATC-3 sample, which may be due to the destruction of the structural integrity of the sample (Figure 3b) caused by the excessive amount of KOH, reducing the efficiency of the ion transport and leading to the decline of its rate capability. When the current density is 0.5 A·g^−1^, the specific capacitance of ATC-4 is 303.1 F·g^−1^, which is higher than that of ATC-2 (281.3 F·g^−1^), but when the current density is above 1 A·g^−1^, its specific capacitance becomes lower than that of ATC-2. At a current density of 10 A·g^−1^, the specific capacitance of ATC-4 decreases to 203.4 F·g^−1^, showing a retention of only 67.1% when compared with the specific capacitance at 0.5 A·g^−1^.

At the same time, from the analysis of the N_2_ adsorption/desorption test, it can be seen that the average pore size of the ATC-4 sample is larger than that of the ATC-3 sample. This may be due to the fact that increasing the volume of pores larger than 2 nm may not have a significant effect on improving the specific capacitance [26]. Although the specific surface area of the ATC-4 sample is larger, its pore structure cannot give full play to the capacitance.

In this study, we also prepared and tested samples converted from tobacco leaves and tobacco stems for comparison. The results (Figure S4) show that the activated carbon materials prepared from tobacco stalks have similar electrochemical properties to those prepared from tobacco leaves and tobacco stems. However, most of the previous studies focused only on tobacco stems. These results prove that tobacco stalks also deserve studying.

In general, the tobacco stem-activated carbon materials obtained in this study have great electrochemical performances, as well as large specific areas. Table 2 shows a comparison of the activated carbon material prepared in some relevant literatures and the ATC-3 sample obtained in this paper. The ATC-3 sample has a larger specific surface area than most of the other activated carbon materials made with KOH as the activating agent and at a similar dosage. The electrochemical properties of the ATC-3 samples are also at a high level, which may prove that tobacco stems are a promising raw material in the field of activated carbon.

### 3.3. The Effect of N, S-Codoping

To further improve the electrochemical performances of the ATC materials, N and S codoping was conducted on ATC-3 samples.

**Figure 5:**
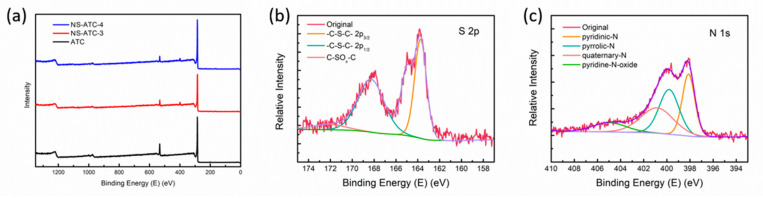
(**a**) XPS spectra of samples before and after N, S-codoping; (**b**) XPS spectra of S 2p of NS-ATC-4; (**c**) XPS spectra of N 1 s of NS-ATC-4.

The element compositions of the samples were characterized by XPS. Two extra peaks could be seen in the XPS spectra of N, S-codoped samples at 166 eV and 400 eV when compared with the spectra of nondoped samples (Figure 5a), which could be attributed to S and N elements, proving the existence of heteroatoms in carbon materials. The content of N and S in the samples was determined by an elemental analysis (Table 2).

As the amount of dopant increases, the N and S content increases from 3.75 wt% and 0.51 wt% for NS-ATC-2 to 5.46 wt% and 0.81 wt% for NS-ATC-4. The oxygen content was also nonignorable. NS-ATC-4 showed an O content of 5.65 wt%, while ATC-3 had an oxygen content of 11.71 wt%, which was higher than most conventional activated carbon materials and indicated the existence of oxygen-containing groups [34].

The binding states of the heteroatom were further investigated by high resolution XPS. The S 1 s spectra (162–170 eV) were composed of several peaks (Figure 5b), including 2p3/2 (163.5 eV) and 2p1/2 (165.0 eV) of C-S-C and C-SOx-C (168.4 eV) caused by the oxidation of C-S-C [35]. The S-doping may lead to a more polarized surface and offer some pseudocapacitance via the reversible reactions listed below, thus increasing the capacitance [36]:

(10) $${-\mathrm{SO}}^{2-}{+2e}^{-}{+H}_{2}O⇌{-\mathrm{SO}}^{-}{+2\mathrm{OH}}^{-}$$

(11) $${-\mathrm{SO}}^{-}{+e}^{-}{+H}_{2}O⇌-S{\left(\mathrm{OH}\right)}^{-}{+\mathrm{OH}}^{-}$$

However, from Table 3 we found that the sulfur content was relatively low, indicating that sulfur introduced via thiourea was not strongly bonded to the activated carbon substrate [37]. EDS mapping was also performed. C, O, N and S elements were distributed evenly in the material (Figure S5).

Meanwhile, the N 1s region could be fitted with four different peaks (Figure 5c): pyridinic-N (N-6, 398.2 eV), pyrrolic-N (N-5, 399 eV), quaternary-N (N-Q, 401.0 eV) and pyridine-N-oxide (N-X, 402.5 eV). N-5 and N-6 could induce more defects and active sites, and N-Q could enhance the conductivity of the materials [38].

**Figure 6:**
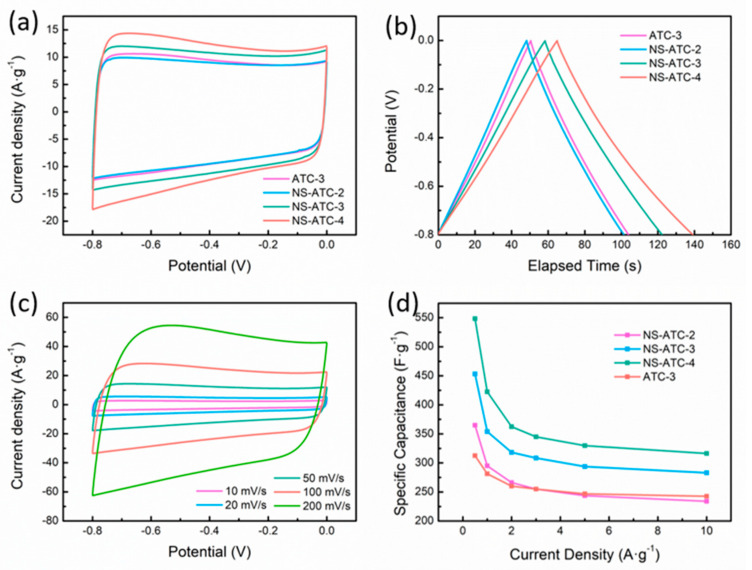
(**a**) CV curves of nondoped and doped samples at 50 mV·s^−1^; (**b**) GCD curves of nondoped and doped samples at 3 A·g^−1^; (**c**) CV curves of NS-ATC-4; (**d**) Specific capacitance of samples at growing current densities.

N, S-codoping endows the samples with a higher specific capacitance. It can be seen that, in the range of this experiment, the CV curve of the doped samples is still an ideal quasi-rectangle (Figure 6a), and there is no obvious redox peak, indicating that the doping of N and S elements did not change the energy storage mechanism of the materials. The GCD curves of the doped tobacco stalk-activated carbon sample have the same characteristics as those of the nondoped sample (Figure 6b), which is consistent with the rectangular shape of the CV curve. However, one can see that the discharge curves of both the nondoped and doped samples show a slight deviation from nonlinearity, and it can be clearly seen that the curves of the N, S-codoped samples are more nonlinear (Figure S6) because the S doping facilitates the HER reaction [39].

By calculating the specific capacitance of the samples according to Equation (1), one can see that, in the range of our experiment, the specific capacitance of the samples increases as the amount of dopant increases, but the capacitance retention goes down. The specific capacitance of the NS-ATC-4 sample at a current density of 0.5, 1, 2, 3, 5 and 10 A·g^−1^ reaches 548.4, 422.5, 362.5, 345.0, 329.7 and 316.3 F·g^−1^, respectively. It is believed that a certain amount of S doping can improve the electrical conductivity of the materials because it could enlarge the interlayer distance of carbon and facilitate the diffusion of ions [40]. However, in this study, it is worth noting that the capacitance retention decreases as the amount of dopant increases (Figure 6d). Compared with the specific capacitance at a low current density (0.5 A·g^−1^), the specific capacitance of the NS-ATC-2/3/4 sample at 10 A·g^−1^ is reduced to 64.1%, 62.5% and 57.7%, respectively. This may be due to the excessive doping destroying the structure of the sample, because S doping produces a more disordered structure and thus reduces the electron transport efficiency. The Raman spectra (Figure S6f) confirm that the I_D_/I_G_ ratio keeps rising with more dopants being used.

The Nyquist curves of the sample also show the same characteristics as those of the nondoped ATC-3 sample. As shown in Figure S6, the curve is nearly vertical in the low-frequency region, and forms a semicircle with a small radius in the high-frequency region.

**Figure 7:**
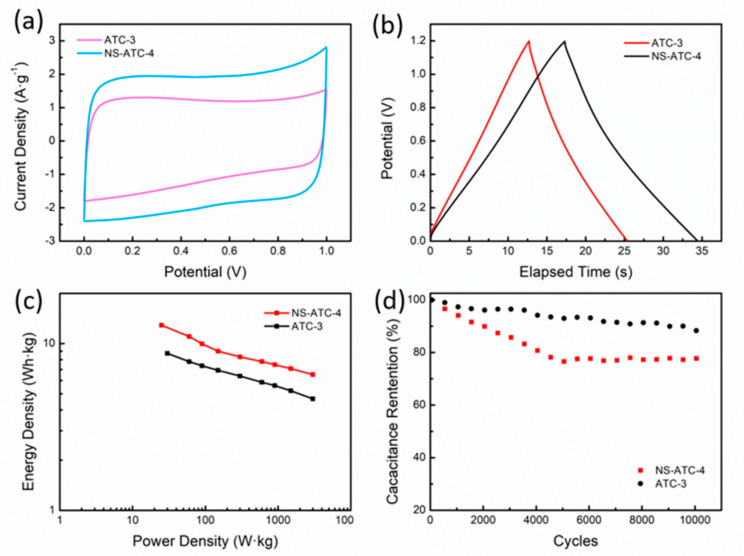
(**a**) CV curves of symmetrical capacitors assembled by ATC-3 and NS-ATC-4 at 50 mV·s^−1^; (**b**) GCD curves of symmetric capacitors assembled by ATC-3 and NS-ATC-4 at 3 A·g^−1^; (**c**) CV curves of NS-ATC-4; (**d**) Capacitance retention of samples after 10,000 cycles of charging and discharging.

Then, ATC-3 and NS-ATC-4 are assembled into solid-state symmetrical supercapacitors to further investigate the electrochemical performance. The CV curves of both ATC-3 and NS-ATC-4 (Figure 7a) show a symmetric rectangular shape, showing the characteristic of a typical double-layer capacitor. The Nyquist plots (Figure S7e) display an approximately vertical line in the low-frequency region and a semicircle with a small radius in the high-frequency region, suggesting a low impedance and fast ion transport. The GCD curves (Figure 7b) are linear without an obvious voltage drop, and the discharge time of NS-ATC-4 is longer than that of NS-ATC-4 under the same current density, indicating a higher specific capacitance. In addition, the specific energy density and power density of the samples are displayed in Figure 7c. The energy density of NS-ATC-4 reaches 12.88 W·h·kg^−1^ at a power density of 25 W·kg^−1^; when the power density is 3000 W·kg^−1^, the energy density is 6.51 W·h·kg^−1^. The cycle life of the sample was also tested (Figure 7d). After 10,000 cycles of charging and discharging at a current density of 5 A g^−1^, the specific capacitance retention of NS-ATC-4 goes down to 77.7%, which is much lower than for the ATC-3 sample (88.3%), indicating a decrease of the cycle stability caused by N, S-codoping.

In general, the results of the electrochemical characterization showed that N and S codoping greatly improved the specific capacitance of ATC materials but also decreased the rate capability and cycle stability. Since the ATC materials showed a high electrochemical performance, they could be used directly without further modification. The method of introducing heteroatoms still needs improvement in order to meet the demands of a long lifecycle.

## 4. Conclusions

This study set out to find a proper method to produce high-performance activated carbon material using tobacco stalks as the carbon source. Samples activated by KOH had ultralarge specific surface areas, and the electrochemical performance of tobacco stem-activated carbon increased at first, before decreasing with an increase in the amount of KOH. The ATC-3 sample showed a high specific capacitance, energy density and power density, as well as a good stability. N and S codoping markedly improved the specific capacitance of ATC materials but also decreased the rate capability and cycle stability. In summary, the results implicate the possibility that tobacco stalks have a great potential to be recycled and used as a raw material for activated carbon materials.

## Figures and Tables

**Table 1:** The content of elements of tobacco stalk ash and TC-550 precursor.

Samples	C (wt%)	O (wt%)	K (wt%)	Cl (wt%)	P	Ca	Mg	Sn
ash	/	49.23	25.17	2.77	1.53	1.27	1.07	0.7
TC-550	90.82	6.50	2.19	0.48	/	/	/	/

**Table 2:** Comparison of the specific surface area and electrochemical performance of biomass-activated carbon materials with ATC-3.

S_BET_ (m^2^·g^−1^)	KOH: Precursor (Mass Ratio)	Specific Capacitance (F·g^−1^)	Electrolyte	Raw Material	Number
1909	2:1	248 (1 A·g^−1^)	6 M KOH	corncob sponge	[27]
2294	6:1	225 (0.5 A·g^−1^)	6 M KOH	sawdust	[28]
655.4	3:1	202 (0.5 A·g^−1^)	6 M KOH	broad beans	[29]
596	2:1	201 (0.5 A·g^−1^)	6 M KOH	graphene	[30]
2757.63	3:1	390 (1 A·g^−1^)	6 M KOH	albizia flowers	[31]
1306	2:1	340 (1 A·g^−1^)	6 M KOH	Human hair	[32]
3326.7	4:1	190 (1 mA·cm^−2^)	1 M LiPF_6_-EC/DMC/DEC	Tobacco stem	[33]
2115	3:1	287 (0.5 A·g^−1^)	6 M KOH	Tobacco stem	[17]
3177	3:1	281.3 (1 A·g^−1^)	6 M KOH	Tobacco stalk	This work

**Table 3:** The content of elements of the samples before and after N, S-codoping.

Samples	C (wt%)	O (wt%)	N (wt%)	S (wt%)
ATC-3	88.29	11.71	-	-
NS-ATC-3	90.47	5.27	3.75	0.51
NS-ATC-4	88.08	5.65	5.46	0.81

## Supplementary Materials

The following are available online at https://www.mdpi.com/2073-4360/12/9/1982/s1. Figure S1. Thermogravimetric analysis of tobacco stalk. Figure S2. CV curves of samples before and after activation. Figure S3. GCD curves and Nyquist plot of ATC-X samples. Figure S4. Electrochemical performance of activated carbon from tobacco leaves and tobacco stems. Figure S5. EDS mapping of NS-ATC-4 sample. Figure S6. Electrochemical performance and Raman spectra of samples with and without doping. Figure S7. Electrochemical performance of symmetrical capacitor assembled by ATC-3 and by NS-ATC-3.
