# Supplementary material for: N, S-Codoped Activated Carbon Material with Ultra-High Surface Area for High-Performance Supercapacitors
Source: Polymers (Basel). 2020 Aug 31;12(9):1982. doi: 10.3390/polym12091982 (PMC7564396; doi:10.3390/polym12091982)
Supplement: Supplementary file 1 [file polymers-12-01982-s001.pdf]

Supplementary Materials

For article " N, S co-doped Activated Carbon Material with Ultra-high Surface Area for High-performance Supercapacitors"

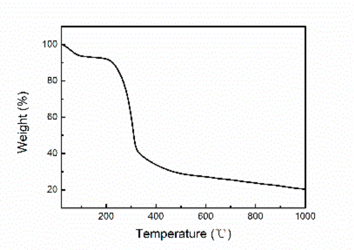

Figure S1. Thermogravimetric analysis of tobacco stalk

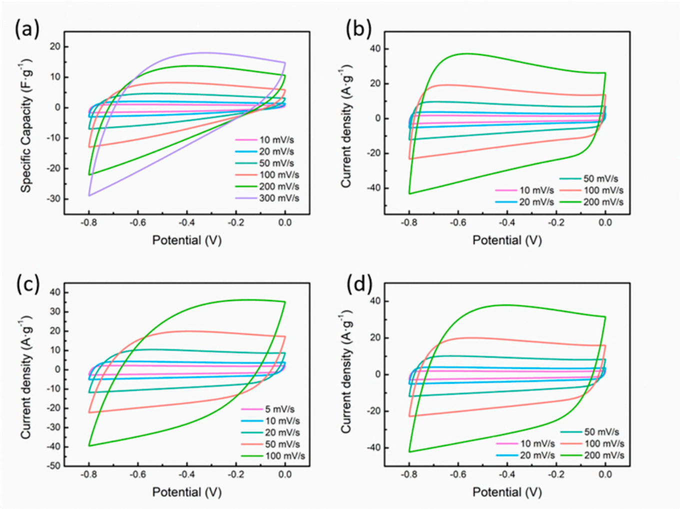

Figure S2. CV curves of (a) TC-850; (b) ATC-1; (c) ATC-2; (d) ATC-3.

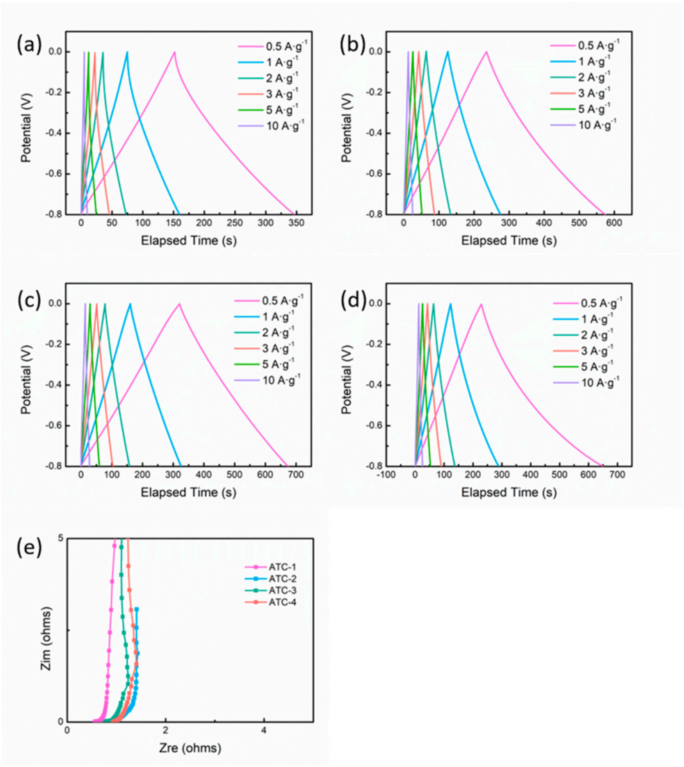

Figure S3. GCD curves of (a) TC-850; (b) ATC-1; (c) ATC-2; (d) ATC-3 and (e) Nyquist plot of ATC-X samples.

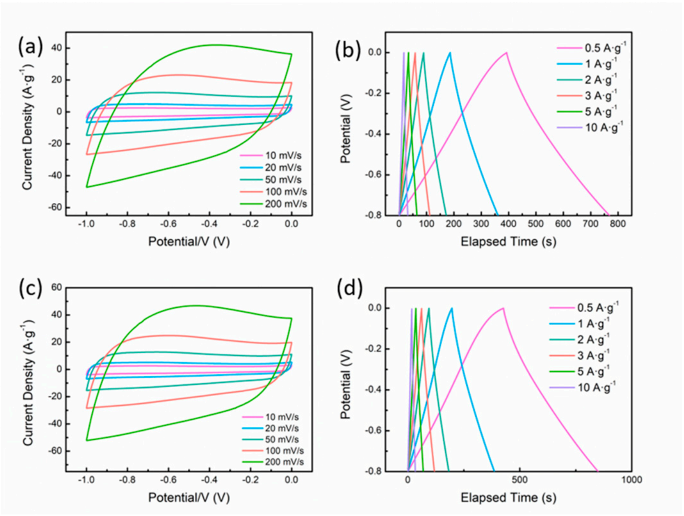

Figure S4. (**a**) CV curves of activated carbon from tobacco leaves; (**b**) GCD curves of activated carbon from tobacco leaves; (**c**) CV curves of activated carbon from tobacco stems; (d) GCD curves of activated carbon from tobacco stems.

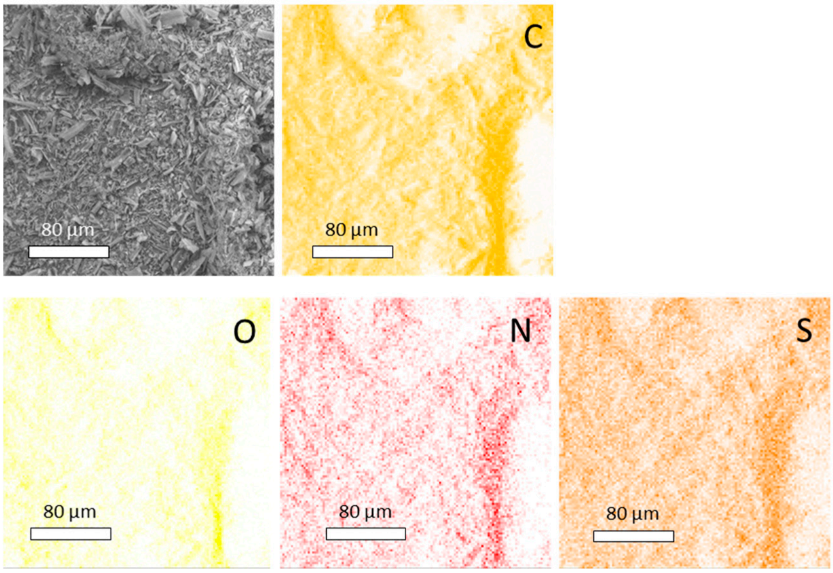

Figure S5. EDS mapping of NS-ATC-4 sample

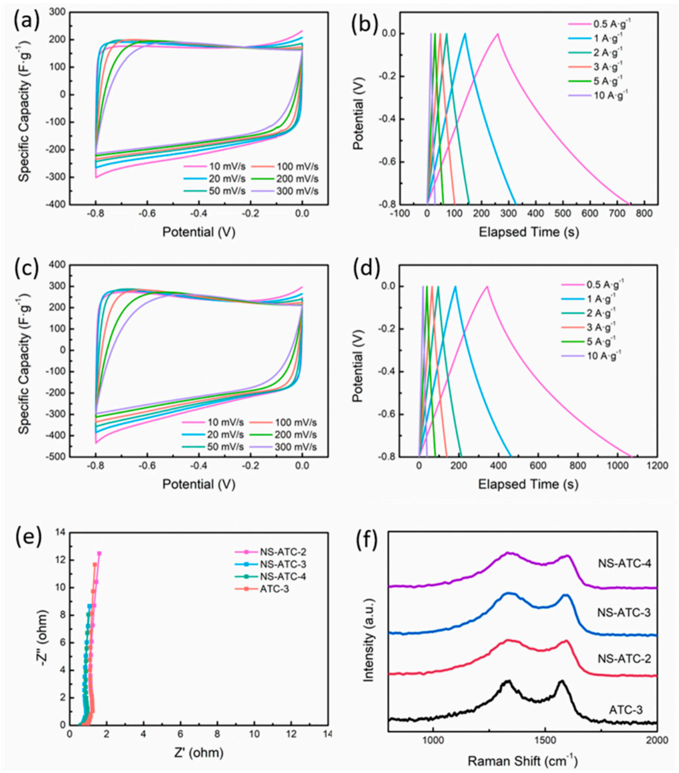

Figure S6. (a) CV curve of NS-ATC-2; (b) GCD curves of NS-ATC-2; (c) CV curves of NS-ATC-3; (d) GCD-curves of NS-ATC-3; (e) Nyquist plot of samples with and without doping; (f) Raman spectra of samples with and without doping.

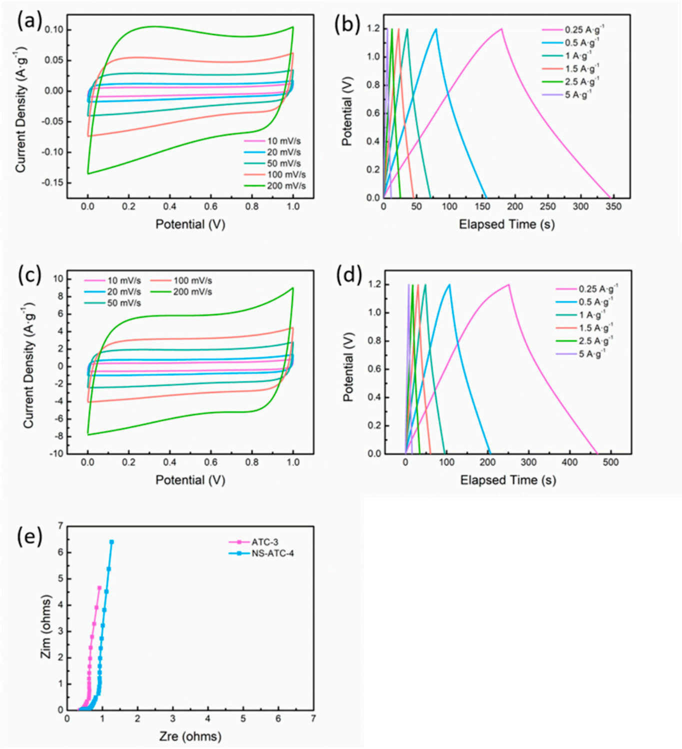

Figure S7. (a) CV curves of symmetrical capacitor assembled by ATC-3; (b) GCD curves of symmetrical capacitor assembled by ATC-3; (c) CV curves of symmetrical capacitor assembled by NS-ATC-3; (d) GCD-curves of CV curves of symmetrical capacitor assembled by NS-ATC-3; (e) Nyquist plot of symmetrical capacitors.
